# Supplementary material for: Effects of menstrual cycle on cognitive function, cortisol, and metabolism after a single session of aerobic exercise
Source: PLoS One. 2024 Oct 29;19(10):e0311979. doi: 10.1371/journal.pone.0311979 (PMC11521275; doi:10.1371/journal.pone.0311979)
Supplement: Attachment — Submitted filename: response to reviewrs organized.docx [file pone.0311979.s003.docx]

| Reviewer | Response |
| --- | --- |
| Title: Effects of menstrual cycle on cognitive function, cortisol and metabolism after a single session of aerobic exercise  Authors: Maryam Mirzaie Khalilabadi, Mohammad Hemmatinafar & Maryam Koushkie Jahromi  Journal: Plos one | We thank the Handling Editor for the opportunity to submit a revised manuscript. We also thank the anonymous Reviewers for their careful and thoughtful comments and critiques.  We believe the reviews helped us to improve the quality of the manuscript.  Please find the detailed point-by-point-response below.  As recommended, all changes **were included in track change. So new pages have been mentioned in revisions.**  Of course, we are ready to do more work on the files, in the event that the revision and the answers are not yet entirely satisfactory. |
| When submitting your revision, we need you to address these additional requirements.  1. Please ensure that your manuscript meets PLOS ONE's style requirements, including those for file naming. The PLOS ONE style templates can be found at  https://journals.plos.org/plosone/s/file?id=wjVg/PLOSOne_formatting_sample_main_body.pdf and  https://journals.plos.org/plosone/s/file?id=ba62/PLOSOne_formatting_sample_title_authors_affiliations.pdf  2. In the online submission form, you indicated that [Data are availbale by corresponding author and will be delivered on request.].  All PLOS journals now require all data underlying the findings described in their manuscript to be freely available to other researchers, either 1. In a public repository, 2. Within the manuscript itself, or 3. Uploaded as supplementary information. | Thank you   1. The manuscript writing was adjusted according to recommendation 2. The only available data is spss data and was uploaded as supplementary information. |
| Reviewer #1: Thank you for such an interesting study. I have a few comments and suggestions, if you don't mind: | Thank you for your encouraging words, devoting your valuable time to reviewing the article. We also appreciate your very important and valuable points that had a great impact on the improvement of the article. |
| 1- In the abstract: would you mind Please to structure the abstract by including opening keywords before each paragraph, such as Aim, Study Design, Results, and Conclusion. | Thank you. Abstract was structured according to recommendation. |
| 2- In the study materials:  - Study setting?  - Would you mind to highlight the type of the study?  - Has any one of the statistical professionals calculated the sample size for you prior to the study? as 12 sample size could be carrying a limitation of the statistical power and increase type 2 error.  - Could you please put a measurable identifications for the different terms you used in the inclusion criteria such as:noramal regular cycle, no obesity , follicular phase, luteal phase.  - You started your study with 12 participants, however you devided them into 5 and 5 in each phasse, any explanation? | Thank you. The following sentences or modifications were included:   - The study setting was Shiraz University which is one of the largest and most important universities in the country, and more than 20000 students from different cities of Iran. - This was a nonrandomized crossover study. - That’s right. Unfortunately we tried as much as possible. However, we could not find any more subject. A method of estimating sample size using G*power was included. - In addition, the small number of participants was included as limitation of the study. - Normal regular cycle, obesity follicular and luteal phases were defined . - Sorry for mistake. It was added. (Introduction and method were provided before the final procedure of experiment and 10 girls predicted to participate while 12 persons participated in final stage. |
| - Would you mind updating some of your references? | Thank you I tried to update references as much as possible and hope it is acceptable. |
| 3- Highlighting strength and limitations of your study would give a realistic impression and would reflect positively on the study application. | Thank you . I tried to complete limitations and applications. |
| 4- The article would benefit from English proofreading as there are several grammatical mistakes. | Thank you. I tried to modify the English writing. |
| Reviewer #2: An interesting and novel study,thank you for the effort done,  I may suggest in future research to recruit a bigger sample and give longer period for follow up ,also to correlate the results with clinical outcome like fertility. | We greatly appreciate your positive and encouraging words.  Thank you for your important suggestion.   - That’s right. Unfortunately we tried as much as possible. However, we could not find any more subject. A method of estimating sample size using G*power was included.   I mentioned them as limitations of the study and surely will use them in my future research. |
| Reviewer #3:  ABSTRACT: The sentence in lines 6-8 should be recasted and grammar corrected. | We greatly appreciate all your suggestions and important points.  Thank you. Abstract was modified. |
| INTRODUCTION: Some grammatical errors that need to be corrected. Page 4, paragraph 1, the last sentence is not clear and should be recasted. | Thank you. Grammar was checked in all parts of manuscript and modifications were considered. |
| METHODS: The authors should state the university where the study was conducted and its location  and also how they determined their sample size.  Page 4, 1st paragraph, line 3, what do the authors mean by active young female students?  The authors should explain how they selected those who participated in the study,  what they mean by - physical and mental health to exercise (page 4, last line),  how they determined those who were obese in order to exclude them.  They should also give examples of the diseases that could affect study variables.  The authors should state the name of the ethics review board that approved the conduct of the study. | - Thank you - The university name was added. - That’s right. Unfortunately we tried as much as possible. However, we could not find any more subject. A method of estimating sample size using G*power was included. - Active women means persons who had a history of participating in sports training regularly at least two sessions a week in the past year.(this sentence was added). - Participants were recruited through announcements in university dormitories and gymnasiums(sentence was added) - Mental and physical health was approved by a physician - The criteria for obesity was mentioned - Examples of disease that could affect the study were added.   _ the name of ethic committee was added. |
| DISCUSSION: There are grammatical errors that make some sentences difficult to understand.  Under strengths and limitations, the last sentence is not clear and should be recasted. | Thank you_grammar was checked in all manuscript and errors were modified.  _ the last sentence was modified . |
| REFERENCES" Some do not appear complete eg numbers 17, 21 and 25 | Thanks References were completed. |
| While revising your submission, please upload your figure files to the Preflight Analysis and Conversion Engine (PACE) digital diagnostic tool, https://pacev2.apexcovantage.com/. PACE helps ensure that figures meet PLOS requirements. To use PACE, you must first register as a user. Registration is free. Then, login and navigate to the UPLOAD tab, where you will find detailed instructions on how to use the tool. If you encounter any issues or have any questions when using PACE, please email PLOS at figures@plos.org. Please note that Supporting Information files do not need this step. | Thanks I tried several times and no change was made on figures by PACE so I submitted in previous format.  If I need to do anything else I greatly appreciate let me know. |
